# Scientific Items

**Published:** 1877-11-20

**Authors:** 


### Scientific Items.

MICA is composed of silex, alumina and potash. There are many varieties. The cleavage of mica is wonderful. Prof. Henry says it may be split into leaves of 250,000 to the inch. It is found in primary formations associated with quartz, etc. It is found in many countries, and is abundant in Middle and North-east Georgia. The uses of mica are various. Diamond dust, with which court dames and our American ladies powder their hair, is ground mica. The costly French silver mouldings are cast from ground mica. The wonderful showers of diamonds I have witnessed in the scenic plays of the "White Fawn" and the "Black Crook," at Niblo's, were mica scales. As a lubricator it is perfection. Mixed with oil it wears longer than any other ingredient. Recent experiments have shown that for any swift running machinery, where the Babbitt metal and other packing have proved at fault, mica packing is perfect; being indestructible by heat, it generates none, and as soon as a good Yankee test is made, the result will be mica-packed boxes for fast, heavy-running machinery, and no more hot boxes or worn journals, being entirely free from grit. For stoves it has now become indispensable, and the demand for clear, transparent mica is rapidly increasing.

---

FIRE.—Some combustible material in a laboratory, at Columbia College, was fired by rays of light concentrated by a globular glass jar filled with water. It is affirmed that, in the forests of Algeria, fires have resulted through drops of water upon leaves, forming lenses.

---

"PHARAOH'S SERPENTS' EGGS are made of sulphocyanide of mercury rendered plastic by the addition of a little mucilage. The salt is best obtained from the manufacturing chemist."—*Ex.*

---

LAVŒSIUM is a new metal named in honor of Lavœsier, and discovered by M. Pratt in Iron Pyrites. It has a silvery white color, malleable and visible.

